# Prevalence and risk factors of mild cognitive impairment and dementia in northern Peru

**DOI:** 10.3389/fpubh.2025.1567073

**Published:** 2025-05-21

**Authors:** Jonathan Zegarra-Valdivia, Leandro Pérez-Fernández, Milagros Casimiro-Arana, Harold Arana-Nombera, Viviana Nayelli Gallegos-Manayay, María del Rosario Oliva-Piscoya, Reyna Alamo-Medina, Eduardo Abanto-Saldaña, Nobuko Vásquez-Zuñe, Lisseth Detquizan Pérez, Diana Gutierrez-Flores, Leslie Lozada Tantarico, Naydelin Hernández, María Celinda Cruz-Ordinola, Carmen Paredes-Manrique, Brenda Chino-Vilca, Gabriel Espinoza, José Cabrejo, Sheila Castro-Suarez, Nilton Custodio

**Affiliations:** ^1^Facultad de ciencias de la salud, Universidad Señor de Sipán, Chiclayo, Peru; ^2^Facultad de psicología, Universidad Tecnológica del Perú, Lima, Peru; ^3^Center of Cognitive and Computational Neuroscience-UCM, Pozuelo de Alarcón, Madrid, Spain; ^4^Achucarro Basque Center for Neuroscience, Leioa, Bizkaia, Spain; ^5^Ministerio De Salud MINSA, Lima, Peru; ^6^Hospital Nacional Almanzor Aguinaga Asenjo ES SALUD Chiclayo, Chiclayo, Peru; ^7^CBI en Demencias y Enfermedades Desmielinizantes del Sistema Nervioso, Instituto Nacional de Ciencias Neurológicas, Lima, Peru; ^8^Atlantic Senior Fellow for Equity in Brain Health at the University of California San Francisco, San Francisco, CA, United States; ^9^Instituto Peruano de Neurociencias, Lima, Peru; ^10^Escuela Profesional de Medicina Humana, Universidad Privada San Juan Bautista, Lima, Peru

**Keywords:** mild cognitive impairment, dementia, risk factors, northern Peru, cognitive decline, prevalence

## Abstract

**Background:**

Global dementia prevalence estimates indicate a growing burden, particularly in Low- and Middle-Income Countries (LMICs). Factors such as education, socioeconomic status, and limited public health interventions contribute to the development of Alzheimer’s disease and other dementias. This study aimed to determine the prevalence of Mild Cognitive Impairment (MCI) and dementia in middle-aged and older adults from northern Peru, as well as identify possible associated risk factors.

**Methods:**

A cross-sectional study was conducted with 385 participants aged 40 to 85 years from Chiclayo, Peru. Cognitive impairment was assessed using the Rowland Universal Dementia Assessment Scale (RUDAS) and INECO Frontal Screening (IFS). Functional activity and depression were evaluated with the Pfeffer Functional Activities Questionnaire (PFAQ) and Patient Health Questionnaire-9 (PHQ-9). Participants were classified as controls, MCI, or dementia based on education-adjusted cutoff scores of those scales through cognitive system classification tools.

**Results:**

According to the results, 31.4% of the sample consisted of subjects without cognitive impairment, 40.5% were identified as possible cases of MCI, and 24.9% as possible cases of dementia. However, the percentages by age group are high. A lower educational level is associated with older age and correlates with lower cognitive scores and functional impairment. Age, hypertension, and hearing loss were significant risk factors for MCI and dementia.

**Conclusion:**

The prevalence of possible MCI and dementia in a city in north Peru is high, with a predominance of MCI and dementia in older adults. Age, low education, hypertension, and hearing loss are potential risk factors for cognitive impairment.

## 1 Introduction

Dementia is increasing significantly in low- and middle-income countries. The Global Burden of Disease Study 2019 projects a sharp increase in dementia cases, estimating that by 2050, the number of affected individuals worldwide will reach 152.8 million, with a significant proportion in LMICs. In particular, the number of people living with dementia in Peru is projected to rise from 196,699 in 2019 to 744,847 by 2050, which will represent a growth of 279% (1).

Over the past 20 years, eight studies about dementia and MCI prevalence have been done in Peru. However, their prevalence estimation has been highly inconsistent, varying from 6.85 to 23.96% in older individuals. One possible explanation is the different methodological approach, making it difficult to know which data are more accurate and limiting appropriate comparisons between populations (2–8). Moreover, two studies report a prevalence estimation of 3.1 and 58.80% for MCI in Perú (9, 10). Another study that assessed the prevalence of possible neurocognitive disorders in 30% included younger (55–59 years) and older adults of the socially and economically vulnerable community (2).

This alarming increase in cognitive impairment has various risk factors that contribute to a high burden of dementia prevalence. In Perú, the overall proportion of dementia cases attributable to these risk factors is estimated to be 44.9% (95% CI: 25.8–61.2%) (11). One study developed in Lima, Perú (2008) found that female gender and low educational level correlated with a high prevalence of dementia (3). Moreover, multimorbidity, particularly the coexistence of type 2 diabetes mellitus (T2DM) and hypertension (HT), has been identified as a significant risk factor for cognitive impairment in this population (12). On the other hand, it is necessary to consider the country’s geographical position and environmental conditions and their impact on population health. According to Urrunaga-Pastor et al. (8), in a systematic review published in 2021, the population who lives in high-altitude has nearly twice the prevalence of MCI and dementia compared to some other regions of the world (8).

Nonetheless, a study that included middle-aged (50–55 years) and older adults in southern Peru found an increase significantly in MCI prevalence with age, reaching 85.5% in individuals aged 86 and older, compared to 34.3% in the 50–55 age group. Sociodemographic factors, such as age, education level, and weekly reading hours, significantly influenced cognitive test performance. These findings underscore the need for targeted public health policies for early detection of MCI, particularly in populations with low educational attainment, to mitigate the progression of the disease and improve cognitive health outcomes (10). Despite the effort being made to investigate the risk factors for dementia, there are still other potentially modifiable factors, such as air pollution, high cholesterol, hearing loss, head injury, obesity, physical inactivity, smoking, social isolation, uncorrected vision loss, excessive alcohol consumption and depression (13), that have not yet been investigated in the Peruvian population.

As mentioned before, there is a need to increase the number of studies that accurately determine the prevalence of MCI and dementia, as well as studies of associated risk factors. This study aims to estimate the prevalence of dementia and MCI in an urban cohort of residents from Chiclayo (north of Peru) and to assess the influence of age, sex, education level, and other relevant factors on cognitive function.

## 2 Methods

**Study design**: This is a two-stage epidemiological study with a quantitative, descriptive, and cross-sectional design in Chiclayo from 2022 to 2024. Chiclayo is located in the north of Peru, on the coast, and is the capital of the Lambayeque department. The National Institute of Statistics and Information (based on the last census of Perú) reported that in 2017, Chiclayo had a population of approximately 799,675 inhabitants (represents the fifth most populated city in Peru), both in urban and rural areas, of which 289,857 inhabitants are over or equal to 40 years of age.

**Participants**: The target population consists of adults aged 40 and older. Of the 400 participants initially evaluated, 385 met the inclusion criteria. The participants in this study did not have any reported history of brain injury, nor were they undergoing neurological or psychiatric treatment. Participation was entirely voluntary, and all individuals provided informed consent. A comprehensive cognitive evaluation was conducted to assess their cognitive functions. Participants were required to be over 40 years old, and there were no restrictions regarding their level of education. We excluded individuals with severe visual or hearing impairments that limited their ability to complete the neurocognitive testing. Data from 372 participants were included in the final prevalence plot, ensuring the exclusion of significant or severe depressive cases to maintain data accuracy.

**Sampling**: To ensure a representative sample, stratified random sampling is employed, considering factors such as gender, socioeconomic level, and area of residence. In the first stage of the study, cluster sampling will be used (Senior Citizen Centers, nursing homes from city districts), considering the available information about the population of adults reported by a government source (14). To calculate the weighted distribution for each district, we also considered the margin of error (5%) and the confidence level (95%). Through this procedure, we will obtain the corresponding for each district to be considered. After the calculations were performed, it was estimated that we would need a sample size of 384 people. Subsequently, in the second stage, random sampling will be conducted in the assigned centers.

### 2.1 Instruments

Sociodemographic and clinical data were collected through structured surveys, which included information on educational attainment, comorbidities, and lifestyle factors. Using a standardized questionnaire, data on age, biological sex, education (including parental education), medical history, and specific mental health conditions were obtained. Additionally, information regarding reading habits and physical activity was recorded.

In line with the latest evidence on dementia risk factors, this study assessed 12 of the 14 modifiable risk factors identified by the Lancet Commission on Dementia (13). These risk factors were evaluated by asking about the past medical history and clinical assessment, and include lower education, hearing loss, hypertension, obesity, smoking, depression, physical inactivity, diabetes, excessive alcohol consumption, traumatic brain injury, high LDL cholesterol, and visual impairment. Although social isolation and air pollution exposure were not explicitly assessed, their potential contribution to dementia risk is known.

To assess the presence of possible dementia and MCI, we administered two brief cognitive tests: Rowland Universal Dementia Assessment Scale (RUDAS) and INECO Frontal Screening (IFS), one instrument for evaluating instrumental activities of daily living (IADLs): Pfeffer Functional Activities Questionnaire (PFAQ) and the depression scale PHQ-9.

**Rowland Universal Dementia Assessment Scale (RUDAS)**: This brief cognitive screening tool isdesigned to minimize the effects of cultural learning and linguistic diversity in assessing initial cognitive performance (15). This study would use the RUDAS-Pe, a Peruvian validation of this scale with good sensitivity and specificity for our population (16). The RUDAS-Pe consists of six components that assess memory, body orientation, visuospatial praxis, motor praxis, judgment, and language for a maximum score of 30.

**INECO Frontal Screening (IFS)**: The current study used the Spanish version (17), validated for a Peruvian population (18). The IFS assesses executive functions, including motor programming, conflict instructions, motor inhibitory control, reverse digit span, verbal working memory, spatial working memory, abstraction, and verbal inhibitory control, for a maximum score of 30.

**Patient Health Questionnaire-9 (PHQ-9)**: It is a standardized, self-administered tool designed to assess the presence and severity of depressive symptoms. Comprising nine items, the PHQ-9 aligns with the diagnostic criteria for major depression as outlined in the DSM-IV and DSM-V. Each item is scored on a scale from 0 to 3, yielding a total score ranging from 0 to 27. The scores categorize depression severity into five levels: no depression (0–4), mild depression (5–9), moderate depression (10–13), moderate–severe depression (14–18), and severe depression (20–27). The PHQ-9 has been validated in Peru with samples from primary care settings and general populations (19, 20), demonstrating high reliability (Cronbach’s alpha >0.80) and sensitivity and specificity exceeding 80% for detecting depressive disorders.

**Pfeffer Functional Activities Questionnaire (PFAQ)**: The questionnaire consists of 10 items that assess abilities such as managing finances, using transportation, preparing meals, making decisions, and managing medications. Each item is scored from 0 to 3, where 0 indicates complete independence, 1 indicates partial dependence, 2 signifies the need for assistance, and 3 indicates complete dependence. The total score ranges from 0 to 30, with higher scores reflecting more significant functional impairment. Studies conducted in Peru have shown the PFAQ to have high reliability (Cronbach’s alpha >0.85) and effectiveness in distinguishing between individuals with mild cognitive impairment and those with dementia (21).

### 2.2 MCI and dementia estimation

To classify individuals with possible MCI and dementia, we employed a two-stage process. Firstly, the RUDAS and the IFS validated cutoff scores for the Peruvian population. The cutoff points were tailored to differentiate cognitive states based on educational level.

For distinguishing between controls and patients with MCI:

***RUDAS*** (22):

- Literate individuals (middle education or higher, approximately 11 years): <24.
- Illiterate individuals (no formal education or less than 6 years): <22.

***IFS*** (22):

- Literate individuals: <24.
- Illiterate individuals: <22.

For differentiating between MCI and dementia:

***RUDAS*** (16):

- Literate individuals: <21.
- Illiterate individuals: <18.

***IFS*** (16):

- For all educational levels: <19.

These thresholds ensure accurate classification across diverse populations, accounting for variations in educational attainment. Adjusting the cutoff points for literacy levels enhances the precision of cognitive assessments in clinical and epidemiological settings. Secondly, we developed a classification system integrating cognitive, functional, and emotional data using standardized clinical variables. This weighted approach generates a composite index, allowing for the categorization of subjects into three primary groups: cognitively unimpaired subjects, MCI, and dementia, while systematically excluding individuals with severe depressive symptoms.

The system utilizes four key measures. The RUDAS assesses overall cognitive status and contributes 40% to the final index, with scores indicating control (2 points), MCI (1 point), or dementia (0 points). The IFS evaluates executive function, weighted at 30%, with the same scoring structure as RUDAS. Functional capacity is measured by the PFAQ, which accounts for 20% of the index. Scores reflect functional independence (0 points) or dependence (1 point) and are inverted to align with the scoring direction of the cognitive measures. Emotional status is evaluated using PHQ-9, with scores of 0 to 2 indicating mild to moderate depressive symptoms (included in the analysis) and scores of 3 or higher indicating severe depression (leading to exclusion). PHQ-9 contributes 10% to the final index.

The composite index (𝑃) is calculated using the formula:


$$\begin{array}{l} P=(\text{RUDAS}\times 0.4)+(\text{INECO}\times 0.3)\\ +((1-\text{Pfeffer})\times 0.2)+((2-\frac{\mathrm{PHQ}}{2})\times 0.1) \end{array}$$

Classification criteria based on the composite score are as follows:

- Cognitively unimpaired subjects: 𝑃 ≥ 1.6
- MCI: 1.2 ≤ *p* < 1.6
- Dementia: *p* < 1.2

Individuals with a PHQ-9 score ≥ 3 are automatically excluded.

It comprehensively evaluates neurocognitive status by incorporating cognitive, functional, and emotional assessments. Additionally, the automatic exclusion of individuals with severe depressive symptoms helps reduce misclassification due to mood-related cognitive impairments, improving diagnostic accuracy. However, its reliance on specific standardized tools (RUDAS, IFS, PFAQ, and PHQ-9) may limit its availability in resource-limited or particular clinical settings.

### 2.3 Procedure

The clinical evaluations and neuropsychological assessments were conducted by a multidisciplinary team composed of neurologists and neuropsychologists with extensive experience in dementia care. These professionals were responsible for ensuring the accuracy of clinical assessments, diagnostic validation, and standardized data collection. Additionally, senior medical and psychology students were trained in administering standardized neuropsychological tests and structured questionnaires. Their training process included theoretical instruction (virtual) and practical sessions under the supervision of experienced clinicians, ensuring adherence to standardized protocols and inter-rater reliability. This training was developed in face-to-face and virtual sessions through four steps: (1) familiarization with instruments, (2) training on the correct administration of all tools (cognitive tests, PHQ-9, and PFAQ), (3) structured sessions with case videos and practice, and (4) evaluation of confirmed cases, comparing their scores. Evaluators whose scores most closely matched the experienced evaluators were selected. This approach ensured high methodological rigor and assessment consistency while optimizing data collection procedures.

A general population screening was conducted to identify potential participants as part of the study procedures. This process was carried out in coordination with the Provincial Municipality, facilitating massive evaluation campaigns across different sectors of the region. Vehicles provided by the university picked up residents in different areas of the city, allowing access to diverse populations, both in urban and rural areas to be evaluated. In addition, assessments were conducted in nursing homes, care centers, and other community spaces, ensuring a broad representation of the target population.

Before participation, all individuals received detailed information about the study’s objectives, procedures, and potential implications. Both verbal and written informed consent were obtained from all participants before the evaluations. Participants were required to sign consent forms to confirm their agreement. In cases where literacy challenges or physical limitations prevented written consent, verbal consent was obtained and formally documented. An authorized caregiver provided their signature when necessary to validate the consent process. This approach ensured that all participants were fully informed and willingly agreed to participate. After obtaining informed consent, participants were interviewed and assessed by trained personnel.

### 2.4 Statistical analysis

To ensure the quality and reliability of the data, we conducted several preliminary analyses before the formal analyses. First, we use descriptive statistics to assess the frequencies, percentages, central tendency, and dispersion measures. Parametric and non-parametric contrast tests (Chi2, Kruskal Wallis H test) were used depending on the normality (checked using Kolmogorov - Smirnov test) and homogeneity of variances (Levene test). Internal consistency was determined using Cronbach’s alpha coefficient (standardized element), and an item-test correlation was used. Furthermore, the age and educational level ranges were evaluated using a one-way ANOVA, finding differences between both variables across the sample. Considering this effect, the second step assessed the differences between the performance on each cognitive test using a Logistic Regression analysis with variables like age, sex, education of the participant and their parents, reading (hours per week), exercise (hours per week), PHQ-9 and PFAQ, adjusting the results for multiple comparisons (Bonferroni correction). The next step of the analysis was to evaluate the Receiver Operating Characteristic (ROC) curve and compare AUCs using the Hanley and McNeil methods. Finally, we obtained the percentage of estimated MCI and Dementia. Statistical analysis was performed with SPSS version 24 (SPSS, Inc., Armonk, NY, United States). Significant results are reported with *p* < 0.05* and *p* < 0.01**.

## 3 Results

Of a total of 400 participants from Chiclayo initially evaluated, only 385 met the inclusion criteria, of which 13 were excluded for major depression (PHQ-9 ≥ 20) for potential misclassification of cognitive impairment, leaving 372 participants. The predominant age group was 50–59 years 117 (30.39%), and the predominant years of study were 13 years and over 143 (37.14%).

The participants were categorized by years of education, ranging from 0 years to 13 or more years, and by age groups (See Table 1). The distribution shows that the proportion of males increases with higher education levels, rising from 21.2% in the group with no formal education to 35.7% in those with 13 or more years of education. Age also varies significantly with education, as those with no education have a mean age of 73.93 years, whereas those with 13 or more years of education have a mean age of 56.43 years. This difference is statistically significant, indicating that younger participants tend to have higher educational attainment.

**Table 1:** Sociodemographic Characteristics illustrating the relationships between education levels and sex, age, cognitive performance, and functional measures.

Education years		0 years		1 – 4 years		5 – 9 years		10 – 13 years		13 or more years		F test	*p* value	
		(*n* = 52)		(*n* = 28)		(*n* = 110)		(*n* = 52)		(*n* = 143)				
Sex	Male	21,20%		14,30%		29,10%		28,80%		35.7%		7.508	0.111	
	Female	78,80%		85,70%		70,90%		71,20%		64.3%				
		M	SD	M	SD	M	SD	M	SD	M	SD			
Age		72,73	12,2	68,75	10,28	62,05	11.29	57,87	8,77	55,63	8,97	32,53	0.000	
Reading (hours/week)		1,83	3,33	2,57	6,97	2,21	3,79	2,37	3,27	3,32	4,37	1,77	0,134	
Exercise (hours/week)		0,73	2,95	0,54	1,58	0,87	1,99	0,37	1,39	1,38	4,38	1,31	0,265	
PHQ-9 Score		6	4,99	5,07	4,66	4,94	4,46	3,54	3,31	3,31	3,56	5,65	0.000	
Functional Activity Pfeffer Questionnarie		6,75	7,97	4,25	7,26	2,06	4,74	0,96	3,23	0,94	1,74	17,63	0.000	
RUDAS		22,98	4,87	24,46	3,26	25,75	3,476	25,85	1,98	26,98	2,69	15,55	0.000	
IFS		12,90	7,49	17,62	5,15	19,30	6,000	22,43	4,33	22,01	3,96	32,80	0.000	
Education (Years)		–	–	3.04	1.48	6.13	2,56	12,08	1.34	13.62	0.97	915,25	0.000	
Father Education (Years)		0.87	2.22	2.57	3.28	3.59	3,71	6,75	4,14	6.83	5.31	25,90	0.000	
Mother Education (Years)		0.90	2.52	2	3.12	2.68	3,45	5,35	3.89	6.18	4.87	24,76	0.000	
Age range		40 to 49 years		50 to 59 years		60 to 69 years		70 to 79 years		80 to 89 years	F test		*p* value	
		(*n* = 67)		(*n* = 117)		(*n* = 107)		(*n* = 67)		(*n* = 27)				
Sex	Male	25,40%		29,90%		31,80%		19,40%		51,90%	10.622		0.031	
	Female	74,60%		70,10%		68,20%		80,60%		48,10%				
Age		44,99	3,28	54,54	2,68	64,08	2,88	73,81	2,8	85,15	4,5	1364,62	0.000	
Reading (hours/week)		2,22	3,54	3,13	5,22	2,64	3,61	1,78	2,94	3,41	5,52	1,49	0,206	
Exercise (hours/week)		0,85	4,42	1,13	3,34	0,93	2,27	0,85	2,94	0,74	2,05	0,16	0,96	
PHQ-9 Score		4,3	4,32	3,82	3,57	3,83	3,95	5,43	4,96	5,41	4,89	2,44	0,047	
Functional Activity Pfeffer Questionnarie		1,12	1,7	0,98	2,59	1,15	2,81	4,82	7,41	9,11	8,83	26,97	0.000	
RUDAS		27,15	2,25	26,64	2,82	26,12	2,76	23,34	4,95	22,93	2,62	20,73	0.000	
IFS		23,09	3,75	21,63	4,37	20,64	4,98	14,37	7,2	13,04	5,46	41,9	0.000	
Education (Years)		10,84	4,47	10,3	4,52	9,36	4,85	4,66	4,64	3,33	4,38	29,63	0.000	
Father Education (Years)		6,31	5,42	6,37	4,93	4,3	4,41	2,24	3,31	2,3	2,92	13,18	0.000	
Mother Education (Years)		5,81	4,91	5,97	4,73	3,4	3,91	1,18	1,95	1,07	1,88	22,83	0.000	

Note: Sociodemographic characteristics of the sample, including comparisons of sex, age, cognitive performance, and functional measures, stratified by education levels. Statistical significance is marked where applicable (*p* < 0.05).

Regarding lifestyle factors, we explored reading and exercise hours; both were more frequent with increasing education. Reading hours per week increased from 1.83 h in the no-education group to 3.32 h in the group with the highest education, while exercise hours increased from 0.73 h to 1.38 h. These trends suggest that higher education is associated with more active and cognitively stimulating lifestyles. Similarly, depression, as measured by PHQ-9 scores, shows a significant association with education; participants with no education have higher depression scores compared to those with more education. Functional independence, assessed by the PFAQ, improves with education levels, where functional impairment is higher in the no-education group (mean score of 6.75) and decreases in those with higher education (mean score of 0.94).

Cognitive performance measured by the RUDAS and IFS tests also shows a clear positive trend with education. Participants with no education have lower RUDAS and IFS scores (22.98 and 12.90, respectively), while those with higher education achieve significantly better scores (26.98 for RUDAS and 22.00 for IFS). These differences highlight the protective role of education in cognitive functioning. When considering age groups, the data show that older participants, particularly those in the 80–89 range, have lower cognitive scores, reduced physical activity, and greater functional impairment than younger groups. For example, functional impairment increases with age, from 1.12 between 40-49 to 9.11 at 80-89 age group.

Using our classification system of estimation of the prevalence of possible cognitive impairment, we found that 31.4% were classified as controls, 40.5% had MCI, 24.9% had dementia, and 3.1% were excluded due to severe depressive symptoms. The data show that male sex is associated with lower odds of MCI (OR = 0.573, 95% CI: 0.338–0.969, *p* = 0.037), indicating that men are less likely to be diagnosed with MCI than women. Among the demographic factors, sex does not show a significant association, with males having an OR of 0.936 (95% CI: 0.506–1.731, *p* = 0.834). Within comorbidities, which are potential risk factors for dementia and MCI, anxiety (mental health disease) has a significant association with dementia, with an OR of 0.293 (95% CI: 0.08–1.07, *p* = 0.05), suggesting a possible protective effect. Hypertension shows a significant association with an OR of 2.918 (95% CI: 1.496–5.689, *p* = 0.001), indicating that individuals with hypertension are almost three times more likely to have dementia. Hypoacusis (hearing loss) is significantly associated with dementia, with an OR of 4.68 (95% CI: 0.949–23.07, *p* = 0.039), suggesting that hearing impairment is a notable risk factor. No significant association was found between MCI and comorbidities; however, hypertension is potentially associated with the disease (OR = 1.77, 95% CI: 0.937–3.342, *p* = 0.076), although it does not reach statistical significance (See Table 2).

**Table 2:** Association between dementia, mild cognitive impairment and comorbidities.

Variables		Dementia	OR	95% CI				MCI	OR	95% CI			
		Observed		Low	High	Chi2	*p* value	Observed		Low	High	chi2	*p* value
	Overall	96						156					
Sex	Male	25	0.936	0.506	1.731	0.044	0.834	57	0.573	0.338	0.969	4.363	**0.037**
	Female	71						99					
Mental health	Anxiety	3	0.293	0.08	1.07	3.838	**0.05**	10	0.622	0.259	1.496	1.146	0.284
	Alcoholism	2	2.553	0.228	28.856	0.62	0.431	6	4.8	0.57	40.414	2.523	0.112
	Depression	11	2.108	0.784	5.663	2.265	0.132	11	1.235	0.46	3.288	0.18	0.673
	PSTD	0	0.553	0.491	0.624	1.602	0.206	3	1.167	0.192	7.094	0.028	0.867
	Drugs Use	1	0.44	0.378	0.511	1.266	0.26	2	0.56	0.504	0.622	1.563	0.211
	Violence	4	0.833	0.228	3.041	0.076	0.782	12	1.597	0.582	4.386	0.838	0.36
Comorbidities	DM	19	1.412	0.695	2.869	0.915	0.339	24	1.04	0.536	2.019	0.014	0.907
	Hypertension	31	2.918	1.496	5.689	10.34	**0.001**	35	1.77	0.937	3.342	3.143	0.076
	Obesity	4	0.541	0.161	1.814	1.017	0.313	6	0.498	0.172	1.439	1.716	0.19
	Cholesterol	12	0.497	0.237	1.043	3.497	0.061	24	0.633	0.344	1.165	2.178	0.14
	Hypoacusia	7	4.68	0.949	23.07	4.281	**0.039**	4	1.566	0.282	8.694	0.267	0.605

Association between dementia, mild cognitive impairment (MCI), and comorbidities. Odds ratios (OR) and confidence intervals (CI) for each variable are presented, highlighting significant risk factors (*p* < 0.05). The bold value indicates statistical significance.

**Figure 1:**
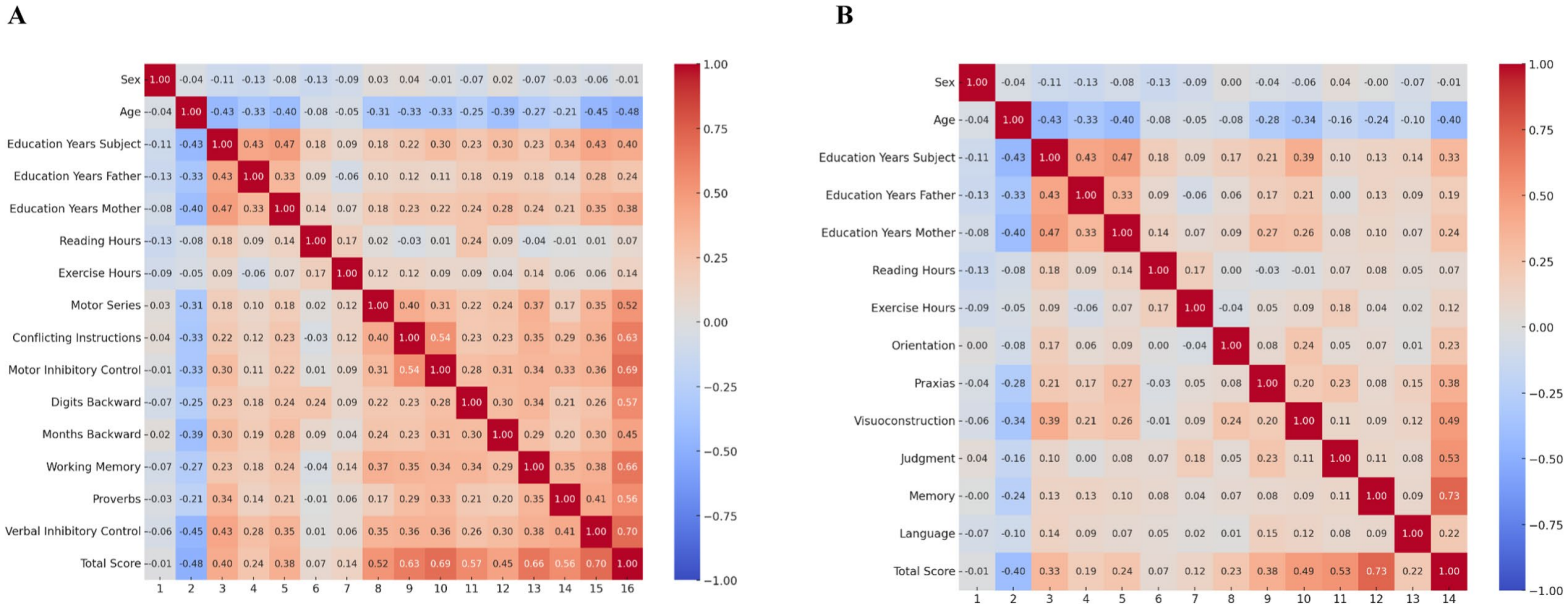
Spearman matrix correlation for cognitive evaluation. **(A)** RUDAS test. **(B)** IFS. The correlations range from −1 to 1, with blue shades representing negative correlations and red shades representing positive correlations.

Figure 1A shows a Spearman correlation matrix illustrating the relationships between demographic factors, functional measures, and cognitive performance in the IFS. Age consistently shows negative correlations with cognitive tasks such as Motor Series (−0.40), Motor Inhibitory Control (−0.33), Working Memory (−0.39), and the Total Score (−0.48), indicating cognitive decline with increasing age. In contrast, education (subject) and mother’s education display positive correlations with cognitive performance, especially with Motor Series (0.43 and 0.36, respectively) and Total Score (0.48 and 0.43).

Exercise hours show modest positive associations with cognitive tasks like Motor Series (0.17) and Total Score (0.14), suggesting potential cognitive benefits of physical activity. The Total Score is strongly correlated with key cognitive tasks, such as Motor Series (0.63), Motor Inhibitory Control (0.60), and Working Memory (0.66). These findings underscore the impact of age, education, and physical activity on cognitive outcomes, highlighting the importance of educational attainment and lifestyle factors in maintaining cognitive health.

Figure 1B presents a Spearman correlation matrix displaying the relationships between cognitive performance, demographic factors, and functional measures with RUDAS. Age shows negative correlations with cognitive tasks, particularly with Total Score (−0.40), Memory (−0.34), Judgment (−0.34), and Visuoconstruction (−0.28). This highlights that increasing age is associated with cognitive decline, especially in memory, judgment, and visuoconstruction abilities.

Education (Subject) is positively correlated with several cognitive domains, such as Visuoconstruction (0.39), Judgment (0.30), and the Total Score (0.33). This indicates that higher educational attainment is linked to better performance across these cognitive tasks. Besides, the mother’s education also shows positive correlations with cognitive tasks, including Judgment (0.29), Visuoconstruction (0.24), and Total Score (0.33), suggesting that early educational environments may influence cognitive performance. On the other hand, reading hours have a modest positive correlation with Orientation (0.18) and exercise hours (0.17), implying that reading habits and physical activity may contribute to maintaining cognitive functions. Exercise hours show a weak but positive correlation with Total Score (0.18), suggesting a potential benefit of physical activity on overall cognitive health. The Total Score is positively correlated with various cognitive tasks, such as Judgment (0.53), Memory (0.73), and Language (0.73), indicating that these domains contribute significantly to overall cognitive performance.

Table 3 presents the results of logistic regression analyses to identify significant covariates influencing performance on the RUDAS and IFS tests. The analyses include two models for each test, displaying coefficients and corresponding *p*-values for each covariate. For RUDAS, age (Model 1: Coefficient = −0.182, *p* = 0.001; Model 2: Coefficient = −0.173, *p* = 0.001), education level (Model 1: Coefficient = 0.123, *p* = 0.027; Model 2: Coefficient = 0.131, *p* = 0.016), and functional activity (Model 1: Coefficient = −0.333, *p* = 0.000; Model 2: Coefficient = −0.353, *p* = 0.000) as significant predictors. These results highlight that age, education, and functional status significantly influence cognitive performance on the RUDAS. In contrast, The logistic regression analysis for the IFS identified age (Model 1: Coefficient = −0.266, *p* = 0.000; Model 2: Coefficient = −0.25, *p* = 0.000), education level (Model 1: Coefficient = 0.190, *p* = 0.000; Model 2: Coefficient = 0.204, *p* = 0.000), mother’s education(Model 1: Coefficient = 2.052, *p* = 0.041; Model 2: Coefficient = 0.099, *p* = 0.03), and functional activity (Model 1: Coefficient = −0.299, *p* = 0.000; Model 2: Coefficient = −0.325, *p* = 0.000), as significant predictors of cognitive performance. These results emphasize that IFS performance is significantly influenced by age, education, maternal education, and functional status, highlighting the importance of these factors in cognitive assessment.

**Table 3:** Logistic regression analysis for confirming covariates involved in the models of cognitive screening tests.

	RUDAS		RUDAS		IFS		IFS	
	Model 1		Model 2		Model 1		Model 2	
	Coeff	*p* value	Coeff	*p* value	Coeff	*p* value	Coeff	*p* value
Age	−0.182	0.001	−0.173	0.001	−0.266	0.000	−0.25	0.000
Sex	−0.002	0.966			−0.016	0.693		
Education (Years)	0.123	0.027	0.131	0.016	0.190	0.000	0.204	0.000
Father education (Years)	0.008	0.878	0.01	0.848	−0.008	0.861	−0.01	0.825
Mother education (Years)	0.011	0.833	0.017	0.739	2.052	0.041	0.099	0.03
Reading (hours/week)	0.011	0.801			−0.001	0.976		
Exercise (hours/week)	0.015	0.736			0.046	0.252		
PHQ-9	−0.052	0.280			−0.065	0.134		
Functional Activity Pfeffer Questionnaire	−0.333	0.000	−0.353	0.000	−0.299	0.000	−0.325	0.000
F	16.884	0.000	30.327	0.000	31.827	0.000	56.524	0.000
Adj R2	0.271		0.276		0.419		0.42	

Logistic regression analysis evaluating covariates associated with cognitive screening tests. Coefficients and *p*-values are provided, emphasizing statistically significant relationships (*p* < 0.05).

**Figure 2:**
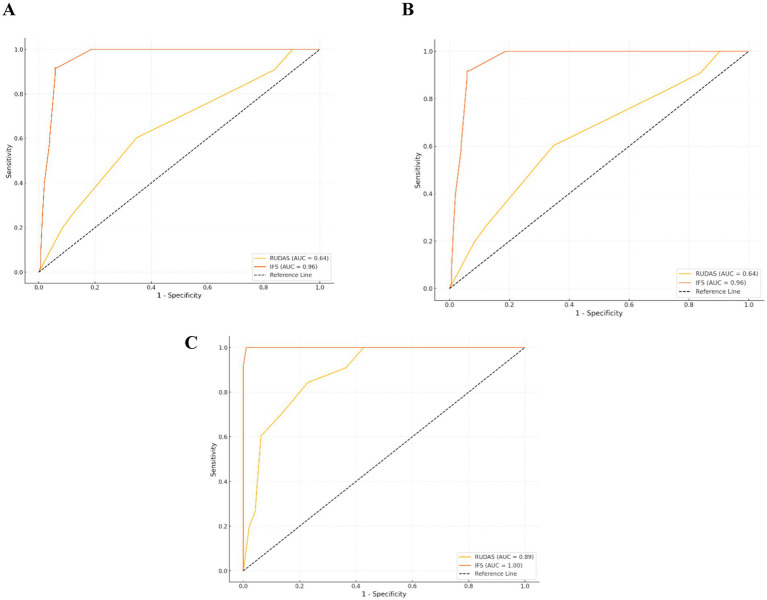
Comparative ROC curves for diagnostic performance across controls, MCI, and dementia groups. **(A)** Controls and MCI group. **(B)** MCI and dementia group. **(C)** Controls and dementia group.

We evaluated the discriminative performance of the RUDAS and IFS tests in distinguishing between individuals diagnosed with MCI and controls, and between MCI and dementia. The analysis employed the ROC curve to measure diagnostic accuracy. The RUDAS test achieved an AUC of 0.64 (95% CI: 0.58–0.71), indicating moderate discriminative ability, while the IFS test demonstrated an AUC of 0.96 (95% CI: 0.94–0.99), reflecting excellent performance in differentiating between groups. The Hanley and McNeil method was applied to statistically compare these AUCs, yielding a difference of −0.32, with a standard error of 0.03, a Z statistic of −10.83, and a *p*-value of 0.001. This significant Z value indicates that the IFS test outperformed the RUDAS test in distinguishing between MCI and controls. These findings suggest that, while both tests have utility, the IFS is significantly more accurate for clinical and epidemiological applications in diagnosing MCI. Figure 2A. On the other hand, the RUDAS test achieved an AUC of 0.82 (95% CI: 0.78–0.87), indicating the good discriminative ability, while the IFS test demonstrated an AUC of 0.85 (95% CI: 0.80–0.90), reflecting similarly good performance in differentiating MCI and dementia. The Hanley and McNeil method was applied to statistically compare these AUCs, yielding a difference of −0.03, with a standard error of 0.02, a Z statistic of −1.02, and a *p*-value of 0.31. This result indicates that the difference between the RUDAS and IFS tests is not significant. Figure 2B. These findings suggest that both tests effectively distinguish between MCI and dementia in clinical and epidemiological applications.

In Figure 2C, we evaluated the discriminative performance of the RUDAS and IFS tests in distinguishing between individuals diagnosed with dementia and cognitively unimpaired subjects. The analysis employed the ROC curve to measure diagnostic accuracy. The RUDAS test achieved an AUC of 0.89 (95% CI: 0.84–0.93), indicating high discriminative ability, while the IFS demonstrated a perfect AUC of 1.00 (95% CI: 0.99–1.00), reflecting optimal performance in differentiating between groups. The Hanley and McNeil method was applied to statistically compare these AUCs, resulting in a difference of −0.11, with a standard error of 0.02, a Z statistic of −5.18, and a *p*-value of (*p* < 0.001). This significant Z value indicates that the IFI test outperformed the RUDAS test in distinguishing between controls and dementia cases. These findings suggest that, while both tests are practical, the IFI test is significantly more accurate for clinical and epidemiological applications in diagnosing dementia.

**Figure 3:**
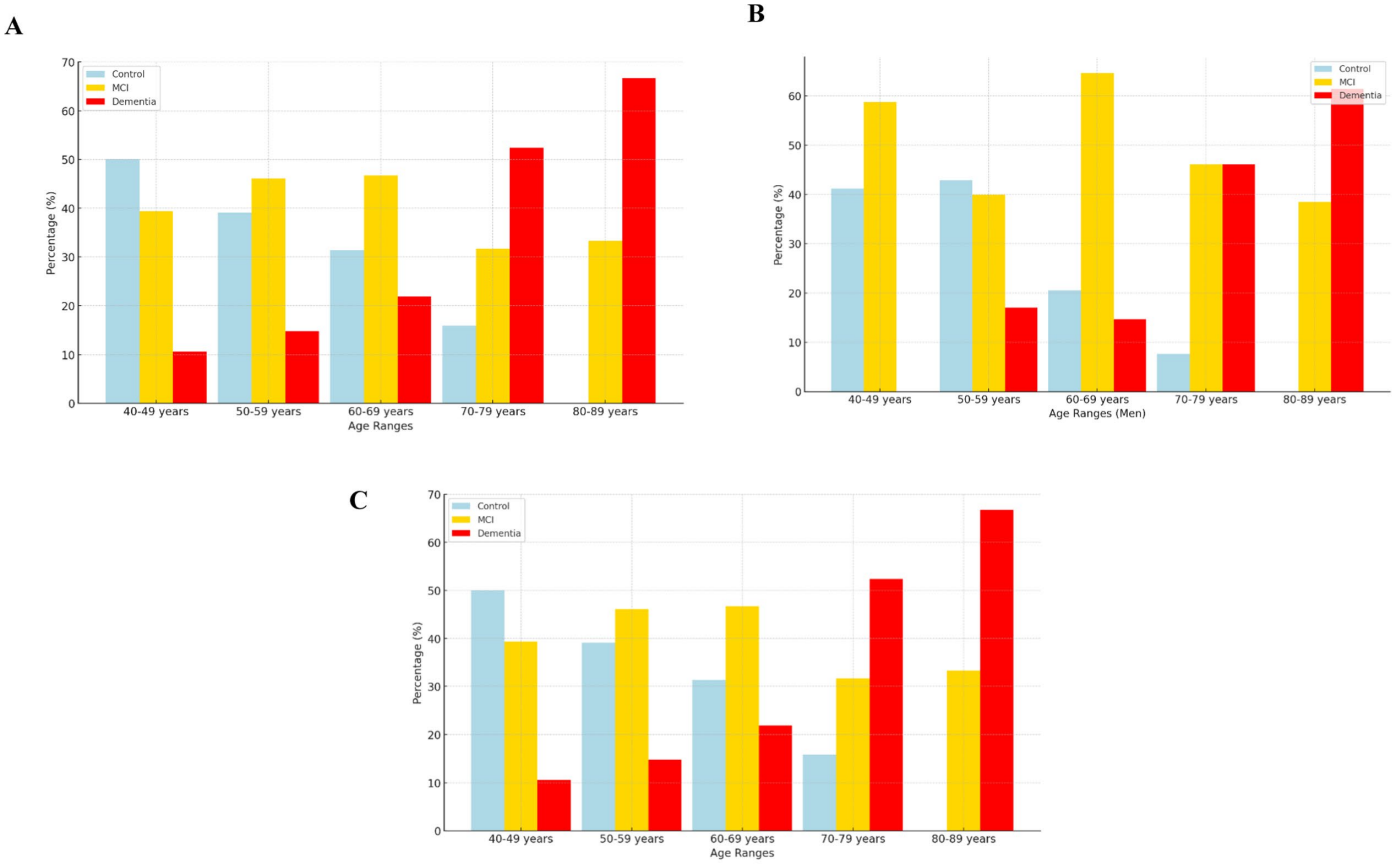
**(A)** Female group. **(B)** Male group. **(C)** Whole group.

Figure 3A shows the distribution of cognitively unimpaired subjects, MCI, and Dementia cases among women across age ranges. Cognitively unimpaired subjects group predominates in the 40–49 age group (50%), but steadily declines by age, disappearing by 80–89 years. MCI remains high in the 50–69 years range (around 47–48%) before decreasing. Dementia prevalence increases sharply after 70 years, peaking at 67% in the 80–89 years group. These trends highlight the importance of early screening and intervention in middle-aged women to mitigate the progression to dementia. Besides, among men, MCI predominates in the 40–69 years groups, peaking at 64% in the 60–69 years range, while controls decline progressively. Dementia prevalence increases sharply after 70 years, reaching 61% in the 80–89 years group. Figure 3B. The decline in controls and the rise in dementia highlight a critical window for early screening and intervention in middle-aged men to delay dementia progression. These findings emphasize the need for targeted cognitive health strategies for aging male populations. Furthermore, considering the distribution of control, MCI, and Dementia cases across age ranges for both sexes, controls dominate in the 40–49 years group (50%) but decline steadily with age, disappearing by 80–89 years. MCI is prominent in the 50–69 years range (around 47–48%), gradually decreasing thereafter. Dementia prevalence increases significantly after 70 years, reaching 67% in the 80–89 years group. Figure 3C. These trends highlight a clear pattern of increasing dementia prevalence with age and underscore the need for early screening and intervention to mitigate cognitive decline in aging populations.

## 4 Discussion

This study is the first prevalence research in northern Peru focused on identifying MCI and dementia using a cognitive system classification tool based on scores from cognitive, depression, and functional scales, as well as to identify potential risk factors in cognitive impairment, providing valuable insights into the cognitive health of this population. The findings of the present study show that about half of those evaluated had possible mild cognitive impairment, and a quarter had dementia. In addition, arterial hypertension and hearing loss are potential risk factors for dementia.

In the study population, the prevalence estimation of dementia is 24.9%. This finding is consistent with a study conducted in 2020 that found a prevalence of dementia of 23.96% in people over 60 years of age (7). However, it differs from two studies that found low prevalence 6.95 and 7.9% in older adults (3, 5). These differences could be due to the methodology used in the studies, one of them was conducted online, and the other two door to door; on the other hand, the assessment instruments differed from those used in this study.

Regarding MCI, the prevalence was 40.5%. A study conducted in southern Peru in 2023 found prevalence figures high and higher than ours (58.8%) in a population aged 50 to 98 years, like ours, but in a highland region (10). Meanwhile, the study conducted by the 10/66 group found prevalence of 3.1% in an urban–rural population of the coast of Peru, like our study population (9). The graphical analysis revealed that the most significant proportion of individuals fell into the MCI category, underscoring the significant burden of cognitive impairment in this population and the potential for early intervention to delay or prevent progression to dementia.

Among the risk factors for dementia, non-modifiable risk factors and modifiable risk factors have been identified. In the present study, the prevalence of MCI and dementia was higher in those older than 50 years and 70 years, respectively. Alike findings have been reported in national and international studies. One study in the capital of Peru found an increase in the occurrence of neurocognitive disorders in 41.8% of younger and older adults (2). Another study conducted in the United Kingdom found that 30.74% of 348 young adults from the United Kingdom have suspected dementia (23). This high prevalence of cognitive compromise in the young adult population should draw the attention of our healthcare systems so that they consider working on preventing modifiable risk factors from the early stages of life.

The present study identified education, arterial hypertension, and hearing loss as possible modifiable risk factors. Our findings reveal that higher educational attainment could be considered a protective factor for dementia. Older age correlates with lower cognitive scores, reduced physical activity, and more significant functional impairment, emphasizing the crucial role of education and lifestyle factors in maintaining cognitive and functional health across the lifespan. It is recognized in the world that people with more childhood education and higher educational levels have a reduced risk of dementia (13). Our results align with previous research highlighting education as a protective factor against cognitive decline and the importance of an active lifestyle in preserving cognitive function. Our study also highlights the likely significant associations between MCI, dementia, hypertension, and hearing loss, consistent with existing international literature (13). These findings emphasize the need for targeted public health strategies prioritizing cardiovascular health management, sensory impairment interventions, and early cognitive screening programs. Addressing these modifiable risk factors could mitigate the burden of cognitive decline and improve health outcomes, particularly in socially and economically vulnerable communities.

### 4.1 Limitations and strengths

This study has several limitations that should be acknowledged. Firstly, the cross-sectional design restricts our ability to infer causal relationships or track the progression of cognitive impairment over time. A longitudinal approach would provide more insight into the incidence of MCI and dementia and the temporal relationship between risk factors and cognitive decline. Secondly, the study relies on specific standardized tools such as the RUDAS, the IFS, the PFAQ, and the PHQ-9. Most commonly used screening tests, such as the MoCA (Montreal Cognitive Assessment) or MMSE (Mini-Mental State Examination), are often significantly influenced by factors such as age, education level, and functional status. In contrast, the tests we selected are less susceptible to these variables and have demonstrated strong sensitivity and specificity in prior studies, making them more reliable tools for distinguishing between dementia, MCI, and healthy controls. While these instruments are validated for the Peruvian population, their availability may be limited in certain clinical or resource-constrained settings, which could hinder the widespread implementation of our classification system.

Another limitation is using weighted scoring for the classification system, which may require weight adjustments when applied to different populations or clinical contexts. The potential for underestimation or overestimation of cognitive impairment classifications underscores the need for further validation across diverse demographic groups. In addition, while we excluded participants with severe depressive symptoms to avoid misclassification, mild to moderate depression was included, which may still influence cognitive performance and introduce some level of bias. Future studies should further investigate the impact of varying degrees of depression on cognitive assessments.

Geographical and sociocultural variability within Peru may also affect the generalizability of our findings. Our study focuses on an urban population in Chiclayo, which may not fully represent rural or high-altitude communities, where factors such as healthcare access, educational attainment, and lifestyle differ significantly. Lastly, although we considered crucial modifiable risk factors like hypertension and hearing loss, our analysis did not include other potential contributors to cognitive decline, such as genetic factors, diet, and environmental exposures. Expanding the scope of risk factors in future research could provide a more comprehensive understanding of cognitive impairment in this population.

Despite these limitations, our study provides valuable insights and is a foundation for future research to improve the diagnosis and management of cognitive impairment and dementia in Peru. Given the significant burden of cognitive impairment identified in this study, public health strategies must prioritize early screening and preventive measures. Integrating routine cognitive assessments into primary care settings could enable the timely detection of at-risk individuals, facilitating early interventions that may delay or mitigate cognitive decline. Furthermore, enhancing access to hearing care and optimizing cardiovascular health management are critical, as hearing loss and hypertension have been consistently recognized as modifiable risk factors for dementia. Public health initiatives should also emphasize educational campaigns to increase awareness about dementia prevention, highlighting the importance of lifestyle modifications such as regular physical activity, cognitive stimulation, and effective vascular health management. On the other hand, incorporating a course on basic health education in elementary school would be important for early identification of risk factors and timely diagnosis and ultimately reduce the economic burden of medical care and long-term care. Implementing these evidence-based strategies in low- and middle-income countries, including Peru, is essential to reducing the growing burden of dementia and fostering cognitive resilience within the population.

In conclusion, the prevalence of possible MCI and dementia in a city in northern Peru is high, with a predominance of MCI in young adults and dementia in older adults. Age, low education, hypertension, and hearing loss are potential risk factors for cognitive impairment. The developed classification of cognitive systems could be considered a robust and integrative tool for identifying neurocognitive status, offering significant potential for epidemiological research, clinical practice, and the design of targeted interventions. By addressing the identified limitations and continuing to refine this system, we can enhance its utility for diagnosing and managing cognitive impairment in Peruvian and similar LMIC populations.

## Data Availability

Data are available upon reasonable request. Further inquiries about the data can be directed to the corresponding author.
